# Nuts as a replacement for carbohydrates in the diabetic diet: a reanalysis of a randomised controlled trial

**DOI:** 10.1007/s00125-018-4628-9

**Published:** 2018-05-23

**Authors:** David J. A. Jenkins, Cyril W. C. Kendall, Benoît Lamarche, Monica S. Banach, Korbua Srichaikul, Edward Vidgen, Sandy Mitchell, Tina Parker, Stephanie Nishi, Balachandran Bashyam, Russell J. de Souza, Christopher Ireland, Sathish C. Pichika, Joseph Beyene, John L. Sievenpiper, Robert G. Josse

**Affiliations:** 1 Department of Nutritional Sciences, Faculty of Medicine, University of Toronto, 5th Floor, Medical Science Building (MSB), 1 Kings College Circle, Toronto, ON M5S 1A8 Canada; 2 Department of Medicine, Faculty of Medicine, University of Toronto, Toronto, ON Canada; 3 Clinical Nutrition and Risk Factor Modification Centre, St Michael’s Hospital, Toronto, ON Canada; 4 Division of Endocrinology and Metabolism, St Michael’s Hospital, Toronto, ON Canada; 5 Li Ka Shing Knowledge Institute, St Michael’s Hospital, Toronto, ON Canada; 6 College of Pharmacy and Nutrition, University of Saskatchewan, Saskatoon, SK Canada; 7 School of Nutrition, Institute of Nutrition and Functional Foods, Laval University, Quebec City, QC Canada; 8 Division of Family and Community Medicine, St Michael’s Hospital, Toronto, ON Canada; 9 Department of Health Research Methods, Evidence, and Impact, McMaster University, Hamilton, ON Canada; 10 Department of Mathematics and Statistics, University of Windsor, Windsor, ON Canada

**Keywords:** Blood lipids, Clotting factors, Glycaemic control, Nuts, Type 2 diabetes

## Abstract

**Aims/hypothesis:**

In line with current advice, we assessed the effect of replacing carbohydrate consumption with mixed nut consumption, as a source of unsaturated fat, on cardiovascular risk factors and HbA_1c_ in type 2 diabetes. The data presented here are from a paper that was retracted at the authors’ request (10.2337/dc16-rt02) owing to lack of adjustment for repeated measures in the same individual. Our aim, therefore, was to fix the error and add new complementary data of interest, including information on clotting factors and LDL particle size.

**Methods:**

A total of 117 men and postmenopausal women with type 2 diabetes who were taking oral glucose-lowering agents and with HbA_1c_ between 47.5 and 63.9 mmol/mol (6.5–8.0%) were randomised after stratification by sex and baseline HbA_1c_ in a parallel design to one of three diets for 3 months: (1) ‘full-dose nut diet’ (*n* = 40): a diet with 2.0 MJ (477 kcal) per 8.4 MJ (2000 kcal) energy provided as mixed nuts (75 g/day); (2) ‘full-dose muffin diet’ (*n* = 39): a diet with 1.97 MJ (471 kcal) per 8.4 MJ (2000 kcal) energy provided as three whole-wheat muffins (188 g/day), with a similar protein content to the nuts, and the same carbohydrate-derived energy content as the monounsaturated fatty acid-derived energy content in the nuts; or (3) ‘half-dose nut diet’ (*n* = 38): a diet with 1.98 MJ (474 kcal) per 8.4 MJ (2000 kcal) energy provided as half portions of both the nuts and muffins. The primary outcome was change in HbA_1c_. The study was carried out in a hospital clinical research centre and concluded in 2008. Only the statistician, study physicians and analytical technicians could be blinded to the group assessment.

**Results:**

A total of 108 participants had post-intervention data available for analysis (full-dose nut group, *n* = 40; full-dose muffin group, *n* = 35; half-dose nut group, *n* = 33). Compared with the full-dose muffin diet, the full-dose nut diet provided 9.2% (95% CI 7.1, 11.3) greater total energy intake from monounsaturated fat. The full-dose nut diet (median intake, 75 g/day) also reduced HbA_1c_ compared with the full-dose muffin diet by −2.0 mmol/mol (95% CI −3.8, −0.3 mmol/mol) (−0.19% [95% CI −0.35%, −0.02%]), (*p* = 0.026). Estimated cholesterol levels in LDL particles with a diameter <255 ångström [LDL-c_<255Å_]) and apolipoprotein B were also significantly decreased after the full-dose nut diet compared with the full-dose muffin diet. According to the dose response, the full-dose nut diet is predicted to reduce HbA_1c_ (−2.0 mmol/mol [−0.18%]; *p* = 0.044), cholesterol (−0.25 mmol/l; *p* = 0.022), LDL-cholesterol (−0.23 mmol/l; *p* = 0.019), non-HDL-cholesterol (−0.26 mmol/l; *p* = 0.020), apolipoprotein B (−0.06 g/l, *p* = 0.013) and LDL-c<_255Å_ (−0.42 mmol/l; *p* < 0.001). No serious study-related adverse events occurred, but one participant on the half-dose nut diet was hospitalised for atrial fibrillation after shovelling snow.

**Conclusions/interpretation:**

Nut intake as a replacement for carbohydrate consumption improves glycaemic control and lipid risk factors in individuals with type 2 diabetes.

**Trial registration:**

ClinicalTrials.gov NCT00410722

**Funding:**

The study was funded by the International Tree Nut Council Nutrition Research and Education Foundation, the Peanut Institute, Loblaw Companies and the Canada Research Chairs Program of the Government of Canada

**Electronic supplementary material:**

The online version of this article (10.1007/s00125-018-4628-9) contains peer-reviewed but unedited supplementary material, which is available to authorised users.

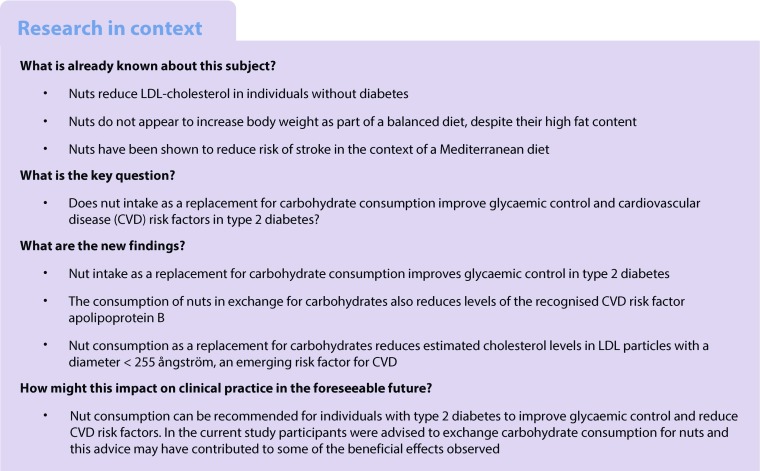



## Introduction

For many years there has been a move towards replacing refined carbohydrates with unsaturated vegetable oils. Cohort studies have indicated benefits for lower carbohydrate diets of plant origin in terms of cardiovascular disease (CVD) and diabetes incidence [1]. Specifically, these benefits have been attributed to the use of nuts [1–4] as a source of vegetable oils. More recently, the benefit of reducing the glycaemic load of the Mediterranean diet by the addition of olive oil or mixed nuts was clearly shown in the PREDIMED (Prevención con Dieta Mediterránea) study, in which 50% of participants had type 2 diabetes. This RCT demonstrated a 30% reduction in CVD risk, especially stroke [5], and a reduction in diabetes incidence [6]. In smaller studies of shorter duration, most types of nuts have also been shown to reduce LDL-cholesterol (LDL-c) [7], improve endothelial function [7, 8] and reduce postprandial blood glucose levels when consumed with a bread-based meal [9]. In the PREDIMED study, a small blood pressure-lowering effect was also observed for both nuts and extra virgin olive oil as compared with a control low-fat diet [10], while it has also been shown that nuts may also be used to treat the metabolic syndrome [11]. As might be predicted, from their LDL-c lowering effects, nuts have been shown to reduce arterial plaque as measured by carotid intima media thickness [12].

As a result of these findings, the US Food and Drug Administration (FDA) released a health claim stating that nuts reduce cardiovascular risk [13]. Moreover, bodies concerned with diabetes and CVD (e.g. the Canadian Cardiovascular Society and the European Atherosclerosis Society) are now advocating increased nut consumption as part of their dietary recommendations [14–17].

We now report the effects of the consumption of mixed nuts in a study of participants with type 2 diabetes that was concluded in 2008 and published in 2011, but retracted in 2016 at the authors’ request because of failure to control for repeated measures in the same individual [18, 19]. Our aim, therefore, is to provide an expanded report that fixes the previous error and adds new data of interest, including data on non-HDL-cholesterol (HDL-c), LDL particle size distribution, and clotting factors VII, VIII, fibrinogen and plasminogen activator inhibitor-1 (PAI-1).

## Methods

### Participants

Participants were recruited by newspaper advertisement and from lists of participants who had taken part in previous studies. Recruitment took place from April 2007 to September 2008, with the last follow-up visit on 18 December 2008. Eligible participants (*n* = 117; see electronic supplementary material [ESM] Fig. 1) were men or postmenopausal women with type 2 diabetes who were taking glucose-lowering agents other than acarbose, and not taking insulin, with medications stable for the previous 3 months. These individuals also had HbA_1c_ levels at screening between 47.5 and 63.9 mmol/mol (6.5% and 8.0%) (Table 1). No participants had clinically significant cardiovascular, renal or liver disease (alanine aminotransferase [ALT] >3 times the upper limit of normal), a history of cancer or were on warfarin. Participants were accepted after surgery or myocardial infarction providing they had an event-free 6 month period prior to the study. One participant changed medications within 3 months prior to the start of the study. Nevertheless, all participants were retained for the analyses.

**Table 1:** Baseline characteristics of study participants

Characteristic	Full-dose nut diet (*n* = 40)	Half-dose nut diet (*n* = 38)	Full-dose muffin diet (*n* = 39)
Age, years	63 (± 8.9)	61 (± 7.9)	61 (± 9.9)
Sex			
Male	26 (65.0)	26 (68.4)	26 (66.7)
Female	14 (35.0)	12 (31.6)	13 (33.3)
Race/ethnicity			
European	23 (58)	25 (66)	18 (46)
Indian	10 (25)	8 (21)	13 (33)
Far Eastern	4 (10)	3 (8)	3 (8)
African	3 (8)	2 (5)	3 (8)
Hispanic	0 (0)	0 (0)	1 (3)
Native American	0 (0)	0 (0)	1 (3)
Weight, kg	80.0 (± 14.7)	86.2 (± 15.6)	82.9 (± 14.7)
BMI, kg/m^2a^	28.8 (± 4.5)	30.3 (± 5.0)	29.4 (± 4.2)
Current smokers	2 (5.0)	4 (10.5)	3 (7.7)
HbA_1c_^b^			
<54.1 mmol/mol (<7.0%)	21 (53)	20 (53)	17 (44)
≥54.1 mmol/mol ≥7.0%)	19 (47)	18 (47)	22 (56)
Duration of diabetes, years	6.7 (± 5.5)	7.9 (± 6.2)	7.9 (± 5.8)
Medication use			
Glucose-lowering medications	40 (100)	38 (100)	39 (100)
Thiazolidinedione	12 (30)	11 (29)	11 (28)
Biguanide	35 (88)	36 (95)	35 (90)
Sulfonylurea	14 (35)	13 (34)	17 (44)
Meglitinides (± non-sulfonylurea)	2 (5)	3 (8)	2 (5)
α-Glucosidase inhibitors	0 (0)	0 (0)	0 (0)
DPP-4 inhibitor	0 (0)	0 (0)	1 (3)
Cholesterol-lowering medications^†^	23 (58)	31 (82)	30 (77)
Blood pressure medications	23 (58)	29 (76)	28 (72)

Data are presented as mean (± SD) or *n* (%)

^a^Calculated as weight in kg divided by height in m^2^

^b^HbA_1c_ <53 mmol/mol (<7%) was used as the therapeutic target [13]

^†^*p* < 0.42, difference between treatments, Fisher’s exact test

DPP-4, dipeptidyl peptidase 4

### Protocol

The study was a 3 month randomised parallel study with two supplements and three treatment groups: (1) ‘full-dose nut diet’: a diet with 2.0 MJ (477 kcal) per 8.4 MJ (2000 kcal) energy provided as mixed nuts (75 g/day); (2) ‘full-dose muffin diet’: a diet with 1.97 MJ (471 kcal) per 8.4 MJ (2000 kcal) energy provided as three whole-wheat muffins/day (188 g/day); (3) ‘half-dose nut diet’: a diet with 1.98 MJ (474 kcal) per 8.4 MJ (2000 kcal) energy provided as half portions of both nuts and muffins (see ESM Table 1). The thermonuclear conversion factor (1 MJ=239.006 kcal) has been used throughout. Further details of the supplements and their dose are given below.

After stratification by sex and HbA_1c_ ≤ 54.1 mmol/mol (≤7.1%), randomisation was carried out anonymously by a geographically isolated statistician. Neither the dietitians nor the participants could be blinded. However, equal emphasis was placed on the potential health benefits of both supplements. The analytical technicians were blinded to treatment, as was the statistician up to and during the preliminary assessment of the primary outcome, HbA_1c_.

Participants were seen at the Clinical Nutrition and Risk Factor Modification Centre in St Michael’s Hospital, a teaching hospital affiliated with the University of Toronto (Toronto, ON, Canada), for screening and assessments at week −1, baseline and weeks 2, 4, 8, 10 and 12 of the intervention. During the first visit, and reinforced at subsequent visits, participants received instruction on how to incorporate the supplement into their diets. At each visit, participants were weighed in indoor clothing without shoes and a fasting blood sample was taken. Only the baseline and final body weight data from the final month of the study were used in the final analysis. Also, at each visit, blood pressure was measured while participants were seated, on three occasions at 1 min intervals, using an OMRON HEM 907 XL automatic sphygmomanometer (OMRON Healthcare, Burlington, Ontario, ON, Canada), and the average of the three measurements was taken. In addition, participants brought 7 day food records to each visit, covering the week prior to the visit; this record was discussed with the dietitian.

During the study, participants were asked not to change their oral glucose-lowering medication use. If participants experienced symptoms of hypoglycaemia with blood glucose levels below 3.5 mmol/l (this occurred in one individual on the full-dose nut diet), and providing hypoglycaemia was not explained by specific circumstances, such as missed meals or increased physical activity, medications were reduced, according to a predetermined protocol, by the participants’ physician. If HbA_1c_ rose above 69.4 mmol/mol (8.5%) on two successive occasions, participants were withdrawn from the study and referred to their own physician. Two participants were withdrawn, one from the full-dose muffin group and the other from the half-dose nut group. Both had HbA_1c_ levels that rose above 69.4 mmol/mol (8.5%) on two successive occasions during recruitment (weeks −1 and 0 for the individual in the half-dose nut group, and weeks 0 and 2 for the individual in the full-dose muffin group (ESM Fig. 1).

The study was approved by the research ethics board of St Michael’s Hospital and the University of Toronto and written consent was obtained from all participants.

### Dietary interventions

General dietary advice conformed to the National Cholesterol Education Program Adult Treatment Panel III (NCEP ATP III) and ADA guidelines. Forty-three per cent of the participants were obese (50/117, BMI>30 kg/m^2^) and wished to lose weight. They were informed that this was not a weight-loss study but were given advice on portion size and food intake to help them meet their body-weight objectives. All participants were advised to reduce total food intake since the supplements replaced approximately 24% of dietary energy, and to take the supplements with meals or as snacks, with a special emphasis on reducing dietary carbohydrate intake. Adherence to the diet supplements was assessed using the final 7 day diet record (obtained at week 12 for *n* = 99 participants or week 8 for *n* = 3 participants for whom week 12 and week 10 diet records were missing) and calculated as a percentage of the amount prescribed.

### Supplements

The nuts supplied consisted of a mixture of unsalted and mostly raw almonds, pistachios, walnuts, pecans, hazelnuts, peanuts, cashews and macadamias provided in equal quantities by weight (donated by the International Tree Nut Nutrition Research and Education Foundation, Davis, CA, USA and the Peanut Institute, Albany, GA, USA). The muffin was whole wheat, sweetened with apple concentrate, with no sugar added (Del’s Pastry, Etobicoke, ON, Canada). The full-dose muffin supplement had a very similar protein content to the full-dose nut supplement, by the inclusion of egg white and skimmed milk powder. The energy from the monounsaturated fatty acids (MUFA) in the full-dose nut supplement was very similar, by design, to the carbohydrate-derived energy in the full-dose muffin supplement (ESM Table 1).

### Energy requirements

Energy requirements were calculated using the Harris–Benedict equation, with allowance for physical activity. Those participants with energy requirements >10 MJ (2400 kcal) were prescribed supplements of 2.6 MJ (630 kcal) (100 g nuts [*n* = 0], 4 muffins [*n* = 1], or 50 g nuts and 2 muffins [*n* = 1]); those whose requirements were 6.7–10 MJ (1601–2390 kcal) were prescribed supplements of 2.0 MJ (477 kcal) (75 g nuts [*n* = 38], 3 muffins [*n* = 36]; or 37.5 g nuts plus 1.5 muffins [*n* = 36]); and those whose requirements were <6.7 MJ (1600 kcal) were prescribed supplements of 1.31 MJ (314 kcal) (50 g nuts [*n* = 2]; 2 muffins [*n* = 2]; or 25 g nuts and 1 muffin [*n* = 1]) (ESM Table 1).

### Biochemical analyses

HbA_1c_ from whole blood, collected in EDTA Vacutainer tubes (VWR International, Mississauga, ON, Canada), was measured within 2 days of collection in the routine biochemistry laboratory of St Michael’s Hospital (Toronto, ON, Canada) by a designated HPLC method (Tosoh G7 Automated HPLC Analyzer, Grove City, OH, USA; CV = 1.7%). Blood glucose was analysed on the day of collection, using a glucose oxidase method on a Beckman Coulter Synchron LX20 analyser (Beckman Coulter, Brea, CA, USA; CV = 1.9%).

Serum samples were obtained from blood which was allowed to clot at room temperature and spun at 2200 *g* for 15 min in a refrigerated centrifuge (Beckman GS-6KR; Beckman Instruments, Palo Alto, CA, USA) and were stored at −70°C and, subsequently, analysed for lipids, apolipoproteins and oxidative lipid and protein products at the end of the study. Total cholesterol (CV = 0.8%), triacylglycerol (CV = 1.2%) and HDL-c (HDLC3 homogeneous assay; CV = 0.9%;) were measured on a Roche Cobas 6000 c501 Analyzer (Roche Diagnostics, Laval, QC, Canada), with LDL-c calculated by the Friedewald equation. Serum lipid measurements were standardised with the CDC Lipid Standardization Program (Centres for Disease Control, Lipid Program, Division of Laboratory Sciences, Atlanta, GA, USA). Apolipoprotein A1 (ApoA1; CV = 1.4%) and apolipoprotein B (ApoB; CV = 2.1%) were measured by nephelometry with the Siemens BN ProSpec analyser (Siemens Canada, Oakville, ON, Canada). C-reactive protein (CRP) was measured by endpoint nephelometry (Siemens BN ProSpec analyser). Oxidised products were only measured in samples from participants who completed the study and had sufficient serum available (*n* = 100). Oxidised LDL-c was measured chemically, as conjugated dienes and thiobarbituric acid reactive substances (TBARS) in the LDL fraction [20] and oxidised serum proteins were measured as protein thiols [21].

The electrophoretic characteristics of LDL-c were assessed by non-denaturing polyacrylamide gradient gel electrophoresis, using serum obtained at baseline and at week 12, stored at −70°C [22]. The relative (%) proportion of each LDL subfraction (LDL size: <255 ångström [Å], 255–260 Å and >260 Å) was multiplied by the total LDL-c level to obtain the cholesterol concentration in each subfraction [22]. Herein, LDL-c_<255Å_ corresponds to the estimated cholesterol levels in LDL particles with a diameter <255 Å.

Clotting factors were measured in plasma collected in citrated Vacutainer tubes at baseline and week 12, stored at −70°C. Levels of clotting factors VII [23], VIII [24] and fibrinogen [25] were measured in the Special Coagulation Laboratory, Hamilton Health Sciences Centre (McMaster Division, Hamilton, ON, Canada) on a STAR/Evolution analyser (Diagnostica Stago, Asnieres Sur Seine, France), using Thombores S reagents (Siemens, Marburg, Germany) for factor VII, Siemens Action FS reagents (Siemens) for factor VIII, and fibrinogen 5 (Diagnostica Stago) for fibrinogen [25]. PAI-1 was measured in the Hemostasis Reference Laboratory, Juravinski Hospital (Hamilton, ON, Canada) [26].

Diets were analysed in the 115 participants with baseline data, using a computer program based on United States Department of Agriculture (USDA) [27] data and international glycaemic index (GI) tables [28], with additional macronutrient, fibre and fatty acid measurements made on local foods, for example speciality breads sold in local supermarkets and the muffins used in this study.

### Power calculation

This study was powered to detect a change in HbA_1c_ of 4.9 mmol/mol (0.45%), similar to a modest effect of acarbose, with an SD of effect for HbA_1c_ of 6.5 mmol/mol (0.60%) [29], under an assumption that allowed for a 25% dropout rate, for which 39 participants per group were required (*α* = 0.05, 1 − *β* = 0.8). The effect size was also in line with the HbA_1c_ data from the completer and intent-to-treat groups from a large low GI study [30]. We therefore aimed to recruit 40 participants per group in our study. The power was designed to assess the primary outcome of the difference in change in HbA_1c_ between the full-dose nut diet vs the full-dose muffin diet, without adjustment for multiple comparisons.

### Statistical analyses

Characteristics of the study participants are expressed as means ± SD for normally distributed variables, or medians and interquartile ranges for variables that were not normally distributed at baseline (Table 2).

**Table 2:** Biochemistry and anthropometric measurements at baseline and study end

	Baseline			Study end		
Variable	Full-dose nut diet (*n* = 40)	Half-dose nut diet (*n* = 38)	Full-dose muffin diet (*n* = 39)	Full-dose nut diet (*n* = 39)	Half-dose nut diet (*n* = 32)	Full-dose muffin diet (*n* = 32)
Glucose, mmol/l^a^	7.17 (1.48)	6.95 (1.22)	7.33 (2.17)	7.20 (1.80)	6.55 (2.90)	7.20 (1.75)
HbA_1c_, mmol/mol^a^	52.6 (7.5)	52.3 (7.3)	53.7 (6.6)	50.8 (9.8)	50.3 (7.7)	52.4 (9.8)
HbA_1c_, %^a^	6.97 (0.68)	6.93 (0.67)	7.07 (0.60)	6.80 (0.90)	6.75 (0.70)	6.95 (0.90)
Total cholesterol, mmol/l^a^	4.10 (1.81)	3.98 (0.93)	4.27 (1.42)	3.96 (1.21)	3.93 (1.06)	4.20 (1.42)
LDL-c, mmol/l^b^	2.46 (1.05)	2.18 (0.60)	2.29 (0.78)	2.27 (1.00)	2.04 (0.61)	2.37 (0.84)
HDL-c, mmol/l^a^	1.12 (0.35)	1.10 (0.31)	1.11 (0.35)	1.23 (0.31)	1.16 (0.34)	1.18 (0.27)
Triacylglycerols, mmol/l^a^	1.43 (0.93)	1.32 (0.64)	1.40 (0.71)	1.35 (1.08)	1.45 (0.93)	1.42 (0.93)
Total cholesterol:HDL-c ratio^a^	3.79 (1.46)	3.39 (0.81)	3.44 (1.39)	3.44 (1.36)	3.27 (1.32)	3.49 (1.69)
LDL-c:HDL-c ratio^a^	1.96 (1.31)	1.86 (0.81)	1.81 (1.27)	1.77 (1.01)	1.73 (1.04)	1.97 (1.28)
Non-HDL-c, mmol/l^a^	3.18 (1.54)	2.78 (0.87)	2.91 (1.47)	3.03 (1.17)	2.73 (1.12)	3.11 (1.55)
Triacylglycerol:HDL-c ratio^a^	1.24 (1.31)	1.11 (0.91)	1.26 (1.15)	1.06 (1.35)	1.29 (1.05)	1.28 (1.05)
ApoA1, g/l^a^	1.43 (0.24)	1.44 (0.37)	1.43 (0.22)	1.43 (0.30)	1.48 (0.35)	1.46 (0.23)
ApoB, g/l^a^	0.84 (0.36)	0.73 (0.21)	0.75 (0.26)	0.78 (0.29)	0.74 (0.24)	0.78 (0.30)
ApoB:ApoA1^a^	0.57 (0.20)	0.50 (0.23)	0.50 (0.25)	0.52 (0.21)	0.44 (0.25)	0.52 (0.28)
CRP, nmol/l^a^	1.04 (1.20)	0.87 (1.69)	1.23 (1.50)	0.86 (1.52)	1.01 (1.73)	0.92 (1.67)
Weight, kg^b^	80.0 (14.7)	86.2 (15.6)	82.9 (14.7)	79.1 (14.0)	84.8 (15.1)	82.6 (14.9)
BMI, kg/m^2 b^	28.8 (4.5)	30.3 (5.0)	29.4 (4.2)	28.5 (4.4)	29.9 (5.2)	29.2 (4.1)
Blood pressure, mmHg						
Systolic^b^	122 (11)	124 (13)	125 (13)	120 (13)	124 (15)	123 (12)
Diastolic^b^	70 (9)	72 (8)	72 (10)	69 (10)	72 (6)	72 (10)
LDL-c<255 Å, mmol/l^a^	1.37 (0.81)	1.28 (0.84)	1.13 (0.80)	1.26 (0.73)	1.14 (0.63)	1.33 (0.90)
LDL-c 255–260 Å, mmol/l^a^	0.50 (0.68)	0.55 (0.36)	0.67 (0.57)	0.49 (0.64)	0.51 (0.37)	0.66 (0.34)
LDL-c>255 Å, mmol/l^a^	0.20 (0.34)	0.30 (0.20)	0.31 (0.48)	0.19 (0.35)	0.27 (0.21)	0.32 (0.37)
Fibrinogen, μmol/l^b^	10.2 (1.3)	10.0 (1.9)	9.4 (1.5)	10.1 (2.0)	9.7 (1.8)	9.0 (1.8)
Factor VII, U/ml^b^	1.15 (0.25)	1.02 (0.28)	1.10 (0.21)	1.13 (0.26)	1.03 (0.23)	1.13 (0.21)
Factor VIII, U/ml^b^	1.08 (0.31)	1.20 (0.38)	1.17 (0.39)	1.20 (0.37)	1.32 (0.46)	1.33 (0.43)
PAI-1, pmol/l^a^	438(434)	470 (227)	475 (438)	392 (215)	496 (368)	404 (381)
Antioxidants	(*n* = 38)	(*n* = 32)	(*n* = 31)	(*n* = 38)	(*n* = 32)	(*n* = 30)
Protein thiols (μmol/l)^b^	317.0 (71.8)	326.6 (72.2)	317.7 (53.8)	355.7 (76.3)	345.9 (6.4)	356.5 (88.7)
Conjugated dienes (μmol/l)^b^	25.2 (9.8)	22.6 (9.8)	24.2 (7.7)	23.0 (9.7)	22.1 (10.0)	21.8 (6.9)
TBARS (μmol/l)^b^	0.32 (0.10)	0.35 (0.12)	0.31 (0.10)	0.32 (0.10)	0.36 (0.12)	0.29 (0.11)

^a^Data are presented as median (interquartile range) for non-normally distributed data

^b^Data are presented as mean (SD) for normally distributed data

For baseline: HbA_1c_, glucose and blood pressure baseline values were calculated as the mean of values at screening and weeks −1 and 0; for weight, BMI and antioxidants, values at week 0 were used; for lipids, lipoproteins and CRP, the mean of weeks −1 and 0 were used (*n* = 93), or screening and week 0 if week −1 was missing (*n* = 17). For *n* = 6, only week 0 data were available. Data were not available for *n* = 1 who was randomised to the half-dose nut group but dropped out prior to week 0; for particle size and clotting factors, week 0 was used (*n* = 113) or week −1 if week 0 had insufficient serum quantities or samples were missing (*n* = 4)

For study end: HbA_1c_, glucose and blood pressure study end values were calculated as the mean of weeks 8, 10 and 12 (final month); for lipid, lipoproteins and CRP, week 8, 10 and 12 samples were also used apart from for *n* = 3 who only had week 8 samples available; for weight and BMI, week 12 values were used (*n* = 100) or, if not available, week 8 values (*n* = 3); for particle size and clotting factors, owing to limited sample availability, week 12 samples were used (*n* = 97) or, if not available, week 10 samples (*n* = 3), or week 8 samples (*n* = 3; where week 10 samples were also unavailable); for antioxidants, week 12 samples were used where serum samples were available (conjugates dienes and TBARS, *n* = 100; protein thiols, *n* = 99 [analytical failure lead to loss of data for *n* = 1 sample])

TBARS, thiobarbituric acid reactive substances

The primary outcome was change in HbA_1c_ from baseline to study end using all available data for an intent-to-treat analysis, acknowledging that nine participants did not have post-intervention data. The primary comparison of interest was the difference in change in HbA_1c_ between the full-dose nut diet and the full-dose muffin diet. The significance of the between-treatment differences was assessed using a repeated measures ANCOVA model, with time treated as a continuous variable (see ESM Methods, SAS code for primary outcome), with a spatial power covariance (PROC MIXED, SAS 9.4; Cary, NC, USA), including all post-intervention time-points as the outcome. The spatial power covariance structure was chosen to account for the within-participant correlation and the unequal spacing of observation times of the repeated measures (at weeks 2, 4, 8, 10 and 12 post-baseline) (the SAS code for the primary outcome is provided in ESM Methods). This covariance structure also yielded the smallest corrected Akaike’s Information Criteria (AICC), indicating that it was the best-fitting model for the observed data.

The Tukey adjustment was applied to the significance levels for pairwise comparisons among the three treatments. No correction was made for the multiple different outcomes assessed (e.g. the 24 biochemical comparisons in Table 3). Application of the Bonferroni correction would require a *p* value <0.0021 (0.05/24) to denote significance. Hence, we regarded these outcomes as exploratory. Covariates included sex and baseline HbA_1c_ (≤54.1 mmol/mol [≤7.1%], >54.1 mmol/mol [>7.1%]), representing stratification factors. Further, lipid medication use at baseline was also a covariate, since it had been determined a priori that variables showing treatment differences at baseline, in this case only lipid medication use (*p* = 0.042), would be adjusted for. Measures that were not normally distributed were transformed using the natural logarithm (log_*e*_) for assessing treatment differences. This process was iterative after assessment of change and not solely based on an examination of baseline information. These adjustments were applied to all measures with the exception of the primary comparison, the difference in change in HbA_1c_ between the full-dose nut diet and the full-dose muffin diet, where the unadjusted significance value is reported in the text. The treatment effect estimates were assessed using the residual maximum likelihood method with the degrees of freedom calculated by the Kenward–Roger method. In the model, treatment, time, the treatment × time interaction, and baseline were treated as fixed effects, while participant was considered as a random effect (ESM Methods, SAS codes for both primary and secondary outcomes).

**Table 3:** Treatment differences in change for blood and anthropometric measurements in the intention-to-treat analysis

	Full-dose nut vs full-dose muffin diet					Full-dose nut vs half-dose nut diet					Half-dose nut vs full-dose muffin diet				
	*β*	95% CI	*p*	Adj CI	Adj *p*	*β*	95% CI	*p*	Adj CI	Adj *p*	*β*	95% CI	*p*	Adj CI	Adj *p*
Glucose (mmol/l)^a^	−0.02	(−0.09, 0.06)	0.610	(−0.11, 0.07)	0.867	−0.05	(−0.12, 0.03)	0.228	(−0.14, 0.04)	0.449	0.03	(−0.05, 0.10)	0.506	(−0.07, 0.12)	0.784
HbA_1c_ (mmol/mol)^b^	−2.0	(−3.8, −0.3)	0.026	(−4.2, 0.1)	0.066	−1.8	(−3.6, −0.04)	0.045	(−4.0, 0.3)	0.111	−0.2	(−2.1, 1.6)	0.813	(−2.5, 2.0)	0.969
HbA_1c_ (mmol/mol)^a^	−0.04	(−0.07, −0.01)	0.017	(−0.08, −0.001)	0.043	−0.03	(−0.07, −0.002)	0.038	(−0.07, 0.01)	0.095	−0.01	(−0.04, 0.03)	0.749	(−0.05, 0.04)	0.945
HbA_1c_ (%)^b^	−0.19	(−0.35, −0.02)	0.026	(−0.38, 0.01)	0.066	−0.17	(−0.33, −0.004)	0.045	(−0.36, 0.03)	0.111	−0.02	(−0.19, 0.15)	0.813	(−0.23, 0.18)	0.969
HbA_1c_ (%)^a^	−0.03	(−0.05, −0.005)	0.018	(−0.06, −0.0002)	0.048	−0.02	(−0.05, −0.001)	0.039	(−0.05, 0.003)	0.097	0.00	(−0.03, 0.02)	0.768	(−0.03, 0.03)	0.953
Total cholesterol (mmol/l)^a^	−0.06	(−0.12, −0.01)	0.026	(−0.13, 0.003)	0.066	−0.04	(−0.10, 0.01)	0.119	(−0.11, 0.02)	0.264	−0.02	(−0.08, 0.04)	0.508	(−0.09, 0.05)	0.785
LDL-c (mmol/L)^a^	−0.09	(−0.18, −0.0004)	0.049	(−0.20, 0.02)	0.120	−0.04	(−0.13, 0.05)	0.383	(−0.15, 0.07)	0.657	−0.05	(−0.15, 0.04)	0.288	(−0.17, 0.06)	0.537
Small LDL-c (<255 Å, mmol/l)^a, c, d^	−0.35	(−0.61, −0.10)	0.007	(−0.66, −0.05)	0.018	−0.07	(−0.32, 0.19)	0.601	(−0.37, 0.24)	0.860	−0.29	(−0.55, −0.02)	0.034	(−0.60, 0.03)	0.085
Medium LDL-c (255-260 Å, mmol/l)^a, c, d^	0.10	(−0.13, 0.33)	0.382	(−0.17, 0.38)	0.655	0.00	(−0.23, 0.24)	0.976	(−0.27, 0.28)	1.000	0.10	(−0.14, 0.34)	0.417	(−0.19, 0.38)	0.694
Large LDL-c (>255 Å, mmol/l)^a, c, d^	−0.08	(−0.52, 0.37)	0.732	(−0.61, 0.46)	0.937	0.18	(−0.27, 0.62)	0.433	(−0.36, 0.71)	0.711	−0.25	(−0.70, 0.19)	0.264	(−0.79, 0.28)	0.502
HDL-c (mmol/l)	0.00	(−0.05, 0.05)	0.987	(−0.06, 0.06)	1.000	0.02	(−0.03, 0.06)	0.472	(−0.04, 0.07)	0.752	−0.02	(−0.07, 0.03)	0.501	(−0.08, 0.04)	0.779
Triacylglycerols (mmol/l)^a^	−0.09	(−0.20, 0.03)	0.159	(−0.23, 0.06)	0.335	−0.11	(−0.23, 0.01)	0.064	(−0.25, 0.03)	0.153	0.03	(−0.10, 0.15)	0.678	(−0.12, 0.17)	0.909
Total cholesterol:HDL-c ratio^a^	−0.06	(−0.13, 0.01)	0.080	(−0.14, 0.02)	0.186	−0.05	(−0.12, 0.02)	0.127	(−0.14, 0.03)	0.277	−0.01	(−0.08, 0.06)	0.825	(−0.09, 0.08)	0.973
LDL-c:HDL-c ratio^a^	−0.09	(−0.19, 0.01)	0.084	(−0.21, 0.03)	0.195	−0.05	(−0.15, 0.05)	0.334	(−0.17, 0.07)	0.598	−0.04	(−0.15, 0.07)	0.460	(−0.17, 0.09)	0.740
Non-HDL-c (mmol/l)^a^	−0.09	(−0.17, −0.01)	0.026	(−0.19, 0.005)	0.067	−0.06	(−0.14, 0.02)	0.114	(−0.16, 0.03)	0.253	−0.03	(−0.11, 0.06)	0.526	(−0.13, 0.07)	0.801
Triacylglycerol:HDL-c ratio^a^	−0.08	(−0.22, 0.06)	0.238	(−0.25, 0.08)	0.464	−0.12	(−0.26, 0.02)	0.085	(−0.29, 0.04)	0.197	0.04	(−0.11, 0.18)	0.607	(−0.14, 0.21)	0.864
ApoA1 (g/l)	−0.03	(−0.08, 0.01)	0.131	(−0.09, 0.02)	0.286	−0.02	(−0.07, 0.02)	0.328	(−0.08, 0.03)	0.590	−0.01	(−0.06, 0.03)	0.604	(−0.07, 0.04)	0.862
ApoB (g/l)^a^	−0.09	(−0.16, −0.02)	0.015	(−0.17, −0.003)	0.039	−0.05	(−0.12, 0.02)	0.176	(−0.13, 0.04)	0.364	−0.04	(−0.11, 0.03)	0.290	(−0.13, 0.05)	0.540
ApoB:ApoA1^a^	−0.06	(−0.14, 0.01)	0.096	(−0.16, 0.03)	0.218	−0.01	(−0.08, 0.07)	0.850	(−0.10, 0.08)	0.981	−0.06	(−0.14, 0.02)	0.155	(−0.15, 0.04)	0.328
Serum CRP (nmol/l)^a^	0.00	(−0.36, 0.36)	0.996	(−0.43, 0.43)	1.000	−0.17	(−0.52, 0.19)	0.359	(−0.59, 0.26)	0.628	0.17	(−0.20, 0.54)	0.375	(−0.28, 0.61)	0.648
Fibrinogen (μmol/l)^a, c, d^	0.04	(−0.04, 0.12)	0.330	(−0.06, 0.13)	0.592	−0.02	(−0.10, 0.06)	0.648	(−0.11, 0.08)	0.891	0.06	(−0.02, 0.14)	0.169	(−0.04, 0.16)	0.352
Factor VII (U/ml)^c, d^	−0.06	(−0.12, −0.002)	0.043	(−0.13, 0.01)	0.106	−0.01	(−0.07, 0.05)	0.715	(−0.08, 0.06)	0.929	−0.05	(−0.11, 0.01)	0.110	(−0.12, 0.02)	0.244
Factor VIII (U/ml)^c, d^	0.02	(−0.08, 0.13)	0.662	(−0.10, 0.15)	0.900	0.01	(−0.10, 0.11)	0.878	(−0.12, 0.13)	0.987	0.01	(−0.09, 0.12)	0.786	(−0.11, 0.14)	0.960
PAI-1 (pmol/l)^c, d^	−45.1	(−178, 87.7)	0.502	(−204, 114)	0.779	−147	(−281, −13.1)	0.032	(−308, 13.6)	0.080	102	(−35.9, 240)	0.145	(−63.4, 268)	0.311
Systolic blood pressure (mmHg)	−0.03	(−3.96, 3.90)	0.990	(−4.73, 4.68)	1.000	−2.84	(−6.75, 1.06)	0.153	(−7.52, 1.83)	0.325	2.82	(−1.27, 6.90)	0.176	(−2.07, 7.70)	0.364
Diastolic blood pressure (mmHg)	−0.68	(−3.05, 1.69)	0.572	(−3.52, 2.16)	0.839	−1.41	(−3.77, 0.95)	0.240	(−4.23, 1.41)	0.467	0.73	(−1.73, 3.19)	0.560	(−2.22, 3.68)	0.829
Weight (kg)^e^	−0.33	(−1.11, 0.45)	0.407	(−1.26, 0.61)	0.683	−0.73	(−1.52, 0.06)	0.070	(−1.67, 0.22)	0.164	0.40	(−0.41, 1.21)	0.330	(−0.57, 1.37)	0.592
BMI (kg/m^2^)^e^	−0.12	(−0.40, 0.16)	0.391	(−0.45, 0.21)	0.666	−0.26	(−0.54, 0.02)	0.070	(−0.59, 0.08)	0.164	0.14	(−0.15, 0.42)	0.343	(−0.21, 0.48)	0.609
Protein thiols (μmol/l)^c, f^	0.42	(−31.2, 32.1)	0.979	(−37.6, 38.4)	1.000	13.0	(−18.7, 44.8)	0.417	(−25.0, 51.1)	0.695	−12.6	(−45.6, 20.4)	0.449	(−52.2, 26.9)	0.728
Conjugated dienes (μmol/l)^a, c, e^	0.09	(−0.07, 0.25)	0.262	(−0.10, 0.28)	0.499	−0.05	(−0.21, 0.11)	0.513	(−0.25, 0.14)	0.789	0.14	(−0.02, 0.31)	0.088	(−0.06, 0.34)	0.202
TBARS (μmol/l)^a, c, e^	0.09	(−0.06, 0.23)	0.247	(−0.09, 0.26)	0.477	0.01	(−0.13, 0.16)	0.865	(−0.16, 0.19)	0.984	0.07	(−0.08, 0.23)	0.339	(−0.11, 0.26)	0.603

Unless otherwise indicated, *n* = 108/117; nine participants (half-dose nut group, *n* = 5; full- dose muffin group, *n* = 4) without post-intervention data are not captured in this analysis

Unless otherwise indicated, the outcome reported is change, modelled as change from baseline. Estimates taken from a repeated measures model in PROC MIXED, SAS 9.4, with sex, binary HbA_1c_ and lipid medications as covariates. Estimated from week 12 using least squares means with Tukey adjusted *p* values and confidence limits from all available data (week 2, 4, 8, 10 and 12)

^a^Data for which residuals were not normally distributed have been log_e_ transformed

^b^No covariates (without sex, binary HbA_1c_ or lipid medications as covariates). Values represent change from baseline, with estimates taken from a repeated measures model in PROC MIXED, SAS 9.4 (as above)

^c^*p* values taken from ANCOVA models; estimates taken from week 12 as outcome model against treatment with sex, binary HbA_1c_ and lipid medication as covariates. Tukey adjustment was applied for pairwise comparisons between the three treatment groups

^d^*n* = 103 (full-dose nut group, *n* = 39; half-dose nut group, *n* = 32; full-dose muffin group, *n* = 32)

^e^*n* = 100 (full-dose nut group, *n* = 38; half-dose nut group, *n* = 32; full-dose muffin group, *n* = 30)

^f^*n* = 99 (full-dose nut group, *n* = 38; half-dose nut group, *n* = 31; full-dose muffin group, *n* = 30); loss of *n* = 1 data point due to analytical failure for one participant in the half-dose nut group

Adj, adjusted; TBARS, thiobarbituric acid reactive substances

Baseline was defined as the mean of available screening values plus weeks −1 and 0 for HbA_1c_, fasting blood glucose and blood pressure variables. Lipids, lipoproteins and CRP were measured in batches in frozen samples; and baseline was calculated as the mean of weeks −1, and 0. If week −1 was missing, the mean of the screening and week 0 sample was used. For other secondary outcomes where samples from both the start and end of the study were available (including LDL particle size and clotting factors), only week 0 and week 12 samples were used for analysis; where week 0 samples were missing, samples from week −1 was used as baseline; where week 12 samples were missing, week 10 samples were used for the end of study measurement or, if these were not available, week 8 samples were used to represent the final month.

Exploratory analyses of the effects of the treatments on dietary variables, LDL particle size, markers of oxidative stress and clotting factors (where only start and end values were available), together with body weight, were all assessed using an ANCOVA with change from baseline (PROC MIXED, SAS 9.4).

A dose–response regression analysis was also carried out using the half-dose nut group as a half dose of the full-dose nut group and assessing outcome as a function of treatment assignment. The model specification was: change in biochemical measure = *α* + *β*_1_ dose + ε_ij_ (where: *α* = *x*-axis intercept; *β*_1_ = slope; dose = 0 for the full-dose muffin group, 0.5 for the half-dose nut group and 1 for the full-dose nut group; and ε_ij_ = residual difference between the observed and predicted value). A single unit increase in the nut dose represented the full dose of nuts prescribed, i.e. 50 g/<6.7 MJ (1600 kcal) per day diet, 75 g/6.7–10 MJ (1601–2390 kcal) per day diet or 100 g/>10 MJ (2400 kcal) per day diet. The changes in biochemical measures (HbA_1c_, lipids and lipoproteins) were calculated from values at the end of treatment (defined as the mean of week 8–12 values) minus the pooled baseline values. The dose–response data were unadjusted and untransformed. Where only a single baseline and a single end of treatment value were available (LDL particle size and clotting factors), these single values were used in the analysis.

## Results

The baseline characteristics of participants are given in Table 1. One participant (1/40) in the full-dose nut group, six participants (6/38) in the half-dose nut group and seven participants (7/39) in the full-dose muffin group dropped out or were withdrawn before the final month of the study (ESM Fig. 1). Allergies developed in two participants: one in the half-dose nut group, and one in the full-dose muffin group. All post-intervention data were retained for the final analyses.

The nutritional profile for each group at baseline and study end are given in ESM Table 2. No differences in diet were seen between treatment groups at baseline. During the study, MUFA intake, expressed as per cent of total energy, increased significantly after full-dose nut consumption (ESM Table 3) compared with the full-dose muffin consumption (9.2% [95% CI 7.1, 11.3]; *p* < 0.001). This change was associated with a reduction in carbohydrate intake. Nuts were eaten in excess of that prescribed by some individuals and so the percentage of recommended nut intake exceeded 100% in some cases. The per cent consumption of prescribed supplements was (mean ± SD): 100.5% ± 12.0% for the full-dose nuts; 105.3% ± 13.7% for the half-dose nuts; 90.7% ± 21.2% for the full-dose muffins.

### Body weight and glycaemic control

In the half-dose nut group, the prescribed dose of oral glucose-lowering medication was increased for one participant during the study, whilst another’s dose was reduced. In the full-dose nut diet, the dose of these therapeutic agents was reduced for two participants. Three participants (one in each group) switched from rosiglitazone (Avandia) to pioglitazone (Actos), following media alerts.

The baseline median HbA_1c_ for the three treatment groups was 52–54 mmol/mol (~7.0%) (Table 2). The primary outcome, change in HbA_1c_, was different in the full-dose nut group compared with the full-dose muffin group, by −2.0 mmol/mol (95% CI −3.8, −0.3 mmol/mol; *p* = 0.026) (−0.19% [95% CI −0.35%, −0.02%]) using a repeated measures mixed model ANCOVA with no adjustment for covariates or multiple comparisons (Table 3). After log_*e*_ transformation for skewed distribution of residuals, the significance levels were somewhat improved (*p* = 0.017) (Table 3). No Tukey-adjusted treatment differences were seen for HbA_1c_ with other comparisons. No statistically significant treatment differences were seen in body weight or blood glucose (Table 3). In the dose–response regression analysis, a 1 unit increase in nut dose (i.e. from 0 to full dose) reduced HbA_1c_ by 2.0 mmol/mol (0.18%) (*p* = 0.044; ESM Table 4).

### Serum lipids and apolipoproteins

One participant in the full-dose nut group increased their lipid medication dose, whilst another on the half-dose nut diet decreased their lipid medication dose during the study. There was no change in lipid medications in the full-dose muffin diet group. Compared with the full-dose muffin diet, the change in ApoB with the full-dose nut diet was significantly different (log_*e*_ ApoB, −0.09 g/l [95% CI −0.17, −0.003]; *p* = 0.039). In the unadjusted model, there was a significant reduction in total cholesterol (−0.06 mmol/l [95% CI −0.12, −0.01]; *p* = 0.026) and LDL-c (−0.09 mmol/l [95% CI −0.18, −0.0004]; *p* = 0.049) with the full-dose nut diet vs the full-dose muffin diet (Table 3). In the dose–response regression analysis, a single unit increase in nut dose reduced total cholesterol (−0.25 mmol/l, *p* = 0.022), LDL-c (−0.23 mmol/l; *p* = 0.019); non-HDL-c (−0.26 mmol/l, *p* = 0.020), and ApoB (−0.06 g/l, *p* = 0.013) (ESM Table 4).

### LDL particle size and clotting factors

A treatment difference was observed in LDL particle size between the full-dose nut group vs the full-dose muffin group (Table 3), with a reduction in LDL-c_<255Å_ particle size distribution (log_*e*_ LDL-c_<255Å_, −0.35 mmol/l [95% CI −0.66, −0.05]; *p* = 0.018). No treatment differences were found in factor VII, factor VIII, fibrinogen or PAI-1, although factor VII was reduced in the full-dose nut group vs the full-dose muffin group using the unadjusted model (−0.06 U/ml [95% CI −0.12, −0.002]; *p* = 0.043). In the dose–response regression analysis, a single unit increase in nut dose reduced LDL-c<_255Å_ (−0.42 mmol/l; *p* < 0.001; ESM Table 4).

### Oxidised LDL-c, plasma proteins, CRP and blood pressure

One participant on the full-dose nut diet had a CRP value of 42.3 nmol/l (the mean of all their other CRP values was 0.72 nmol/l, ± 0.15), and this high CRP value was excluded from all the analyses as it was an extreme outlier. No other data from this participant were excluded. No significant differences in CRP, blood pressure, or measures of oxidative damage were seen between treatments (Table 3).

### Adverse events

One serious adverse event requiring hospitalisation was reported by a participant on the half-dose nut diet who developed atrial fibrillation while shovelling snow and was hospitalised for 6 days. Additionally, the following adverse events resulted in withdrawal or dropout prior to the final month of the trial (ESM Fig. 1): in the full-dose nut group, one participant was advised not to eat nuts because of abdominal pain, later diagnosed as being related to *Helicobacter pylori* infection; in the half-dose nut group, one participant had a possible nut allergy; in the full-dose muffin group, one participant developed diarrhoea that resolved after discontinuing muffin consumption, another participant developed allergic symptoms related to the muffins and one participant suffered from leg swelling that resolved after Avandia (rosiglitazone) medication was discontinued.

## Discussion

Increased mixed nut consumption favourably affected HbA_1c_, LDL-c and ApoB levels. These data provide a specific food option for those with type 2 diabetes wishing to lower the carbohydrate content of their diet.

In 1994, the ADA first suggested the possibility of exchanging dietary carbohydrate for MUFA in dietary recommendations for type 2 diabetes [31]. Since then, many [5, 32] although not all, studies have shown beneficial effects of MUFA in diabetes [32]. In 2011, our own study demonstrated that the use of nuts as a replacement for carbohydrates in the diet led to an improvement in glycaemic control in type 2 diabetes [18]. However, we have withdrawn that paper because of our failure to adequately account for repeated measures in the same participant. Our current manuscript still demonstrates the glycaemic advantage of nuts and also shows a potential CVD advantage in reduced LDL-c, ApoB and LDL-c<_255Å_ in the dose–response regression analysis of nuts. In the PREDIMED study, MUFA in the form of either extra virgin olive oil or nuts, reduced CVD in high-risk participants, especially atrial fibrillation and stroke [5]. In the current study, participants were instructed to reduce their intake of carbohydrates, especially of starchy foods, to accommodate the muffin and nut supplements. No significant overall weight change was observed with any of the treatments. Consumption of nuts has not been associated with increased body weight despite their high lipid content. Assessing energy intake in those who were on the full-dose nut diet or full-dose muffin diet, a significant change in energy intake was only observed in those on the full-dose nut diet with a BMI over 30 kg/m^2^; in these individuals, there was a 0.87 MJ (208 kcal) increase (*p* = 0.042) in energy intake, but with no significant increase in body weight (0.37 kg, *p* = 0.452), possibly reflecting the reduced bioavailability of nut lipids [33].

Cohort studies have provided additional support for the association of higher vegetable fat and protein intake with reduced risk of developing diabetes and CHD [34, 35]. More specifically, nut consumption has been shown to be associated with reduced CVD risk, total cancer and all-cause mortality [36].

The relative reduction in HbA_1c_ in this study was small (2.0 mmol/mol [0.19%]). Nevertheless, this reduction was approximately half that recognised by the FDA as being therapeutically significant for new drugs (3.3–4.4 mmol/mol [0.3–0.4%], assuming a fasting level of 54.1 mmol/mol [7.1%] for the calculation) [37]. Moreover, this beneficial effect of nut consumption on HbA_1c_ was seen in those already treated with one to two (average, 1.5) glucose-lowering medications and despite 44–53% of the participants in the three diet groups having baseline HbA_1c_ levels already at target (Table 1) [14]. The potential added benefit of nuts on HbA_1c_ may result from a number of factors that may affect postprandial blood glucose. First, nuts added to a meal will reduce the glycaemic load of the meal if isoenergetic intake is maintained. Second, the presence of lipids together with antinutrients, such as phytates and antioxidant phenolics, may delay gastric emptying and the rate of small intestinal absorption, resulting in a flatter postprandial glycaemic response [9].

Pharmacological interventions aimed at improving glycaemic control must have no negative impact on CHD risk. In our study, increased nut consumption not only improved glycaemic control but was also negatively associated with lipid risk factors for CHD. The significance of LDL particle size on CHD risk is still debated but evidence from the Quebec Cardiovascular Study suggests a strong positive association between LDL-c_<255Å_ and CHD risk [22, 38]. Small LDL particles may have pro-atherogenic properties compared with large LDL particles, owing to increased vulnerability to oxidative damage [31]. Most studies show that isoenergetic exchange of carbohydrate for fat tends to reduce serum levels of small LDL particles [39], although others have not observed this effect [40]. In our study, the change in lipid and lipoprotein risk factors for CVD (LDL-c, LDL-c_<255Å_, non-HDL-c, and ApoB) were negatively related to the changes in nut intake, supporting the recognised hypocholesterolaemic effects of nuts [7] and providing additional mechanistic evidence for the association of nut intake with reduced CVD risk [5, 39, 41].

Higher circulating clotting factor VII concentrations have also been associated with increased CHD risk. This has been demonstrated in the Northwick Park Heart Study [42] and, more recently, in the Prospective Cardiovascular Münster (PROCAM) study [43], in which the association between raised clotting factor VII and the incidence of CHD was assessed at the respective 5 and 8 years follow-up of these cohorts. In the PROCAM study, in 1780 healthy men, 130 events occurred during follow-up, with a significant increase (~3.4%) in factor VII levels when all CHD events were considered [43]. Perhaps for reasons of power, nut consumption did not have an effect on clotting factors in our study; although a significant change in factor VII was observed when using the unadjusted comparison of the full-dose nut diet vs the full-dose muffin diet (*p* = 0.043), significance was lost after adjustment for multiple comparisons.

The major weakness of the study is that it was underpowered to establish a dose response to nuts. Also, in the present study, nut consumption was substantial (75 g/8.4 MJ (477 kcal), equating to 24% of energy intake) for the full-dose nut diet. However, compliance levels were high: 100.5% and 105.3% for the full-dose nut and half-dose nut groups, respectively.

Further a study-wide adjustment was not made for the multiplicity of comparisons. The secondary outcomes must therefore be seen as exploratory. Since this is a post-retraction reanalysis of a previously published trial, all results could be considered post hoc. In terms of the original protocol, insulin, HOMA-IR, serum amyloid A, IL-6 and waist and hip circumference were not measured, but LDL particle size, non-HDL-c, factor VII, factor VIII, fibrinogen and PAI1 were measured as emerging risk factors for CVD. By comparison with the retracted study, we have now lost significance for total cholesterol, LDL-c, total cholesterol:HDL-c, LDL-c:HDL-c, and ApoB:ApoA1 in comparison of the full-dose nut diet with the full-dose muffin diet. Significance was also lost for HbA_1c_, total cholesterol and systolic and diastolic blood pressure when the full-dose nut group was compared with the half-dose nut group. Nonetheless, significance was retained for the primary outcome, HbA_1c_, and also for ApoB, and it was gained for LDL-c_<255Å_, when comparing the full-dose nut group with the full-dose muffin group.

The statistical approach used in the current study represents a departure from the pre-specified original analytical plan. The statistical analysis was originally intended to involve *t* tests and χ^2^ tests, together with standard equation modelling. Use of a repeated measures mixed model (ANCOVA) was also originally considered as an option to account for possible confounders. We believe our current approach fulfils the original intention by appropriately accounting for the correlation between repeated measures in the same individual and the use of covariates to adjust treatment effects.

Finally, our failure to demonstrate a treatment difference in the antioxidant effects of nut consumption may relate to the relatively high content of antioxidants in wheat bran and apple concentrate used in the muffins.

The strengths of the study include its novelty as one of the few studies to assess the effects of the consumption of mixed nuts in type 2 diabetes. It is also one of very few studies to assess the effects of nut consumption on apolipoproteins and the only study to examine the effect of nuts on LDL particle size and clotting factors. Another strength of the study was that supplement compliance was good.

In conclusion, the exchange of carbohydrate intake in the form of a whole-wheat muffin for monounsaturated fats in nuts improved glycaemic control in diabetes and was negatively associated with lipid risk factors for CVD. These data support the benefit of including nuts in the diet of individuals with diabetes despite previous concerns over their high fat and energy density.

## Electronic supplementary material

ESM (PDF 255 kb)

## Data Availability

The authors believe in the principle of making data freely available, but this was not anticipated or specifically approved by the St Michael’s Hospital Research Ethics Board at the time the study was initiated. However, specific requests received by the authors for data sharing for purposes such as data verification and meta-analyses will be considered by the St Michael’s Hospital Research Ethics Board on an individual basis, under defined and mutually agreed-upon conditions.

## Abbreviations

Å: Ångström
ApoA1: Apolipoprotein A1
ApoB: Apolipoprotein B
CRP: C-reactive protein
CVD: Cardiovascular disease
FDA: US Food and Drug Administration
GI: Glycaemic index
HDL-c: HDL-cholesterol
LDL-c: LDL-cholesterol
LDL-c_<255Å_: Estimated cholesterol levels in LDL particles with a diameter <255 ångström
MUFA: Monounsaturated fatty acids
PAI-1: Plasminogen activator inhibitor-1
PREDIMED: Prevención con Dieta Mediterránea
PROCAM: Prospective Cardiovascular Münster (study)
