# Supplementary material for: Nuts as a replacement for carbohydrates in the diabetic diet: a reanalysis of a randomised controlled trial
Source: Diabetologia. 2018 May 23;61(8):1734–47. doi: 10.1007/s00125-018-4628-9 (PMC6061153; doi:10.1007/s00125-018-4628-9)
Supplement: Supplementary file 1 — (PDF 255 kb) [file 125_2018_4628_MOESM1_ESM.pdf]

ESM Methods

SAS code for Primary outcome

Code is bolded and explanations are asterisked.

```
proc mixed method = reml data = dm_nut;
                *Mixed model, residual maximum likelihood estimation method;
        where week in (2,4,8,10,12);
                *All available data used;
        class rand week id ;
        model hba1c100_chg = time|rand/ddfm=kr(firstorder);
                *hba1c100_chg = change from baseline post-intervention data;
                *time = week, continuous;
                *rand*time = allows variation by time;
                repeated week /subject=id type=sp(pow)(time);
                *Models serial correlation by correlating errors, spatial structure for unequal timepoints;
        lsmeans rand /diff cl at time = 12;
                *Takes estimates from end of study;
run;
```

SAS code for secondary outcomes (adjusted for stratification factors (sex and hba1c100>7.1) and baseline lipid medications with descriptions Code is bolded and explanations are asterisked)

```
proc mixed method = reml data = dm_nut;
                *Mixed model, residual maximum likelihood estimation method;
        where week in (2,4,8,10,12);
                *All available data used;
        class rand week id sex_num dichohba1c100;
        model secondary_chg = time|rand sex_num dichohba1c100 lipid_meds/ddfm=kr(firstorder);
                *secondary_chg = change from baseline post-intervention data;
                *time = week, continuous;
                *rand*time = allows variation by time;
                *sex_num = 0-Male and 1-Female;
                *dichohba1c100 = 0-baseline hba1c100 < =7.1 and 1- baseline hba1c100 >7.1;
                *lipid_meds =  lipid medication use;
        repeated week /subject=id type=sp(pow)(time);
                *Models serial correlation by correlating errors, spatial structure for unequal timepoints;
        lsmeans rand /diff cl at time = 12  adjdfe = row adjust = tukey;
                *Takes estimates from end of study, Tukey adjustment for multiple comparisons;
run;
```

ESM Table 1 Amounts and Nutritional Analysis of Prescribed Supplements per day

Treatment and Dose [a]	Total Participants	Energy MJ (kcal)	% energy [c]	Total Fat (g)	MUFA (g)	PUFA (g)	SFA (g)	Dietary Cholesterol (mg)	Total Protein (g)	Plant Protein (g)	Total Carb (g)	Total Fibre (g)	Soluble Fibre (g)	Available Carb (g)	Omega 3 (g)	Omega 6 (g)
Full Dose Nuts																
>10 MJ = 100g nuts	0	2.66 (636)	<26.5	58.3	32.9	16	6.8	0	16	16	20.1	8.4	1.4	11.7	1.3	14.7
6.7-10 MJ = 75g nuts	38	2.00 (477)	19.9-29.8	43.8	24.7	12	5.1	0	12	12	15	6.3	1.1	8.8	1	11
<6.7 MJ = 50g nuts	2	1.33 (318)	>19.9	29.2	16.4	8	3.4	0	8	8	10	4.2	0.7	5.9	0.7	7.3
Half Dose Nuts																
>10 MJ	1	2.65 (633)	<26.3	43.2	18.2	16	6.8	1.3	16	12.9	53.1	8.4	1.5	44.8	0.7	15.3
6.7-10 MJ	36	1.98 (474)	19.8-29.6	32.4	13.6	12	5.1	1	12	9.6	39.9	6.3	1.1	33.6	0.5	11.5
<6.7 MJ	1	1.32 (316)	>19.8	21.6	9.1	8	3.4	0.7	8	6.4	26.6	4.2	0.8	22.4	0.4	7.7
Muffins [b]																
>10 MJ = 4 muffins	1	2.63 (629)	<26.2	28.1	3.5	16	6.8	2.6	16	9.7	86.2	8.4	1.6	77.9	0	16
6.7-10 MJ = 3 muffins	36	1.97 (471)	19.6-29.4	21.1	2.6	12	5.1	2	12	7.3	64.7	6.3	1.2	58.4	0	12
<6.7 MJ = 2 muffins	2	1.31 (314)	>19.6	14	1.7	8	3.4	1.3	8	4.9	43.1	4.2	0.8	38.9	0	8

[a] > 10MJ (>2400 kcals); 6.7-10MJ (1600 – 2400 kcals); and 6.7MJ (<1600 kcals).

[b] 1 muffin = 62.5g.

[c] The full dose nut therefore provided 23.9% of energy on an 8.4 MJ diet.

ESM Table 2 Nutritional Profiles at week 0 and week 12 for Participants Consuming Full Nut Dose, Half Nut & Muffins

	Week 0			Week 12		
	Nuts (N=39)	Half dose (N=37)[a]	Muffin (N=39)	Nuts (N=39)[b]	Half dose (N=32)	Muffin (N=32)
Energy (MJ)[c]	7.8(3.3)	7.7 (2.7)	7.7 (2.9)	8.5(2.0)	8.2 (2.6)	7.6 (2.8)
Energy (kcal)[c]	1863 (780)	1839 (641)	1837 (694)	2024 (483)	1960 (621)	1821 (676)
Total fat (% energy)[d]	32 (7)	33 (6)	34 (6)	42 (4)	39 (5)	36 (4)
MUFA (% energy)[d]	12.8 (3.5)	13.4 (2.6)	13.4 (3.5)	19.6 (2)	15.8 (2.8)	11.4 (2.6)
PUFA (% energy)[d]	6.6 (2.1)	6.7 (2.1)	6.9 (2.1)	9.5 (1.7)	9.8 (1.7)	10.4 (2.1)
SFA (% energy)[d]	9.9 (2.7)	10.2 (2.9)	10.7 (3)	9.4 (2)	10.1 (2.3)	10.7 (1.8)
Dietary Cholesterol (mg/MJ)[c]	0.64 (0.26)	0.55 (0.21)	0.62 (0.41)	0.48 (0.28)	0.54 (0.32)	0.48 (0.22)
Dietary Cholesterol (mg/kcal)[c]	153 (63)	132 (49)	148 (98)	114 (67)	130 (76)	115 (53)
Total protein (% energy)[d]	20 (4)	20 (4)	20 (3)	18 (3)	19 (3)	20 (4)
Plant Protein (% energy)[c]	7 (3)	7 (2)	7 (3)	8 (2)	8 (2)	7 (2)
Available Carbohydrate (% energy)[d]	45 (10)	45 (8)	44 (8)	39 (7)	41 (6)	44 (6)
Starch (% energy)[c]	27 (12)	30 (8)	28 (9)	12 (7)	13 (5)	14 (7)
Sugar (% energy)[d]	16 (6)	14 (4)	15 (5)	25 (5)	27 (5)	28 (5)
Fibre (g/MJ)[c]	0.06 (0.02)	0.06 (0.02)	0.06 (0.03)	0.06 (0.01)	0.06 (0.02)	0.06 (0.02)
Fibre (g/kcal)[c]	15 (5)	15 (6)	13 (8)	14 (3)	15 (5)	14 (4)
Alcohol (% energy)[c]	0 (3)	0 (2)	0 (3)	0 (3)	0 (1)	0 (2)
Glycemic Index[c]	78 (7)	80 (9)	79 (5)	75 (9)	79 (6)	81 (6)
Glycemic Load[c]	114 (52)	120 (66)	106 (60)	106 (43)	116 (57)	119 (45)
Nuts (g/day)[c]	6 (17)	10 (21)	5 (17)	75 (5)	38 (10)	0 (3)
Supplements (% energy)[d]				24 (5)	26 (8)	25 (8)

a Also in the half nut dose diet one participant dropped out before providing a baseline diet record.

b One full dose nut participant had no baseline diet record and a further full dose nut participant dropped out before completion. Therefore only 38 participants diet records on the full dose nut diet were available to calculate change in dietary intake.

c Data are medians (interquartile ranges) for non-normally distributed data.

d Data are means (standard deviation) for normally distributed data.

ESM Table 3 Treatment Differences in change for energy, nutrients, fiber, and supplement intake in the Intention-to-Treat Analysis (n=108/117)[a]

	Full Nut vs Muffin					Full Nut vs Half Nut					Half Nut vs Muffin				
	β	95% CI	*p*	Adj CI	Adj *p*	β	95% CI	*p*	Adj CI	Adj *p*	β	95% CI	*p*	Adj CI	Adj *p*
Energy (MJ)	0.62	(-0.18, 1.42)	0.128	(-0.34, 1.59)	0.28	0.06	(-0.76, 0.87)	0.891	(-0.92, 1.03)	0.99	0.57	(-0.26, 1.4)	0.179	(-0.43, 1.56)	0.368
Energy (kcal)	148.8	(-43.7, 341.4)	0.128	(-82.1, 379.8)	0.28	13.4	(-180.7, 207.5)	0.891	(-219.3, 246.2)	0.99	135.4	(-62.9, 333.8)	0.179	(-102.4, 373.3)	0.368
Total fat (% energy)	8.38	(5.07, 11.69)	<.0001	(4.41, 12.35)	<.0001	3.19	(-0.14, 6.53)	0.06	(-0.81, 7.19)	0.144	5.19	(1.78, 8.6)	0.003	(1.1, 9.27)	0.009
MUFA (% energy)	9.2	(7.5, 10.9)	<.0001	(7.1, 11.3)	<.0001	4.3	(2.6, 6.1)	<.0001	(2.2, 6.4)	<.0001	4.9	(3.1, 6.7)	<.0001	(2.8, 7)	<.0001
PUFA (% energy)	-0.41	(-1.56, 0.74)	0.484	(-1.79, 0.97)	0.763	-0.5	(-1.66, 0.66)	0.392	(-1.89, 0.89)	0.667	0.09	(-1.09, 1.28)	0.874	(-1.33, 1.52)	0.986
SFA (% energy)	-0.47	(-1.64, 0.7)	0.427	(-1.87, 0.93)	0.705	-0.48	(-1.66, 0.7)	0.421	(-1.89, 0.93)	0.699	0.01	(-1.2, 1.21)	0.987	(-1.44, 1.45)	1
Dietary Cholesterol (mg/ MJ)	-0.06	(-0.17, 0.05)	0.306	(-0.2, 0.08)	0.561	-0.11	(-0.22,0.01)	0.063	(0.25, 0.03)	0.15	0.05	(-0.07, 0.17)	0.402	(-0.09, 0.19)	0.678
Dietary Cholesterol (mg/kcal)	-14	(-41, 13)	0.306	(-47, 19)	0.561	-26	(-54, 1)	0.063	(-59, 7)	0.15	12	(-16, 40)	0.402	(-22, 46)	0.678
Total protein (% energy)	-1.66	(-3.32, -0.004)	0.0495	(-3.65, 0.33)	0.12	-1.12	(-2.8, 0.55)	0.185	(-3.13, 0.88)	0.38	-0.54	(-2.25, 1.17)	0.533	(-2.59, 1.51)	0.806
Plant Protein (% energy) [a]	0.13	(0.02, 0.24)	0.02	(0, 0.26)	0.051	0.03	(-0.08, 0.14)	0.597	(-0.1, 0.16)	0.857	0.1	(-0.01, 0.21)	0.078	(-0.03, 0.23)	0.181
Available Carbohydrate (% energy)	-6.5	(-10.3, -2.7)	0.001	(-11.1, -2)	0.003	-2.3	(-6.1, 1.6)	0.241	(-6.9, 2.3)	0.468	-4.2	(-8.1, -0.3)	0.035	(-8.9, 0.5)	0.087
Starch (% energy)	-3.36	(-6.42, -0.3)	0.032	(-7.03, 0.31)	0.08	0.28	(-2.81, 3.36)	0.86	(-3.42, 3.97)	0.983	-3.63	(-6.78, -0.48)	0.024	(-7.41, 0.15)	0.062
Sugar (% energy)	-3.14	(-5.4, -0.88)	0.007	(-5.85, -0.43)	0.019	-2.55	(-4.83, -0.27)	0.029	(-5.28, 0.18)	0.072	-0.59	(-2.92, 1.74)	0.617	(-3.38, 2.2)	0.87
Fibre (g/ MJ) [a]	-0.12	(-0.25, 0.02)	0.087	(-0.28, 0.04)	0.2	-0.08	(-0.22, 0.05)	0.229	(-0.24, 0.08)	0.449	-0.03	(-0.17, 0.1)	0.624	(-0.2, 0.13)	0.875
Fibre (g/ kcal) [a]	-0.12	(-0.25, 0.02)	0.087	(-0.28, 0.04)	0.2	-0.08	(-0.22, 0.05)	0.229	(-0.24, 0.08)	0.449	-0.03	(-0.17, 0.1)	0.624	(-0.2, 0.13)	0.875
Alcohol (% energy) [a]	-0.08	(-0.76, 0.6)	0.812	(-0.9, 0.74)	0.969	0	(-0.69, 0.69)	0.996	(-0.84, 0.84)	1	-0.08	(-0.74, 0.58)	0.81	(-0.88, 0.72)	0.968
Glycemic Index [a]	-0.09	(-0.16, -0.02)	0.008	(-0.17, -0.01)	0.021	-0.04	(-0.1, 0.03)	0.247	(-0.12, 0.04)	0.477	-0.05	(-0.12, 0.02)	0.137	(-0.13, 0.03)	0.296
Glycemic Load	-16	(-31, -1)	0.047	(-34, 3)	0.114	-6	(-22, 9)	0.428	(-25, 12)	0.706	-9	(-25, 6)	0.242	(-28, 10)	0.469
Nuts (g/day) [a]	2.87	(1.92, 3.81)	<.0001	(1.73, 4)	<.0001	0.92	(0.29, 1.56)	0.005	(0.16, 1.68)	0.014	1.94	(0.99, 2.9)	0.0001	(0.8, 3.09)	0.0004
Supplements (% energy)	-1.47	(-4.77, 1.83)	0.378	(-5.43, 2.48)	0.651	-3.42	(-6.7, -0.14)	0.041	(-7.36, 0.52)	0.102	1.95	(-1.49, 5.39)	0.263	(-2.17, 6.07)	0.501

Outcome is change, modelled as change from baseline. Estimates taken from week 12 using least squares means with Tukey adjusted *p*-values and confidence limits, from a repeated measures model using PROC MIXED of SAS 9.4, with sex, binary HbA1c and lipid medications as covariates.

a Log_e transformation of data for which residuals were not normally distributed.

ESM Table 4 Dose Response Regression Model for the blood and particle size*

	β	*p*
HbA1c (%)	-0.18	0.044
HbA1c (mmol/mol)	-2.0	0.044
Total cholesterol (mmol/l)	-0.25	0.022
LDL cholesterol (mmol/l)	-0.23	0.019
HDL cholesterol (mmol/l)	0.01	0.618
Triacylglycerols (mmol/l)	-0.07	0.461
Total cholesterol: HDL ratio	-0.22	0.083
LDL: HDL ratio	-0.20	0.057
Non-HDL cholesterol	-0.26	0.020
Triacylglycerols: HDL ratio	-0.04	0.681
ApoA1	-0.02	0.403
ApoB	-0.06	0.013
ApoB:apoA	-0.03	0.088
Small LDL (< 255 Å, mmol/l)	-0.42	0.0003

* p-values taken from the dose-response regression models. The change in biochemical measures (HbA_{1c}, lipids and lipoproteins) was calculated as values at the end of treatment (defined as the mean of week 8–12 values) minus the pooled baseline values. Total n= 103 (39 nut, 32 half nut, 32 muffin).

ESM Figure 1:
Flow of Participants through the Trial

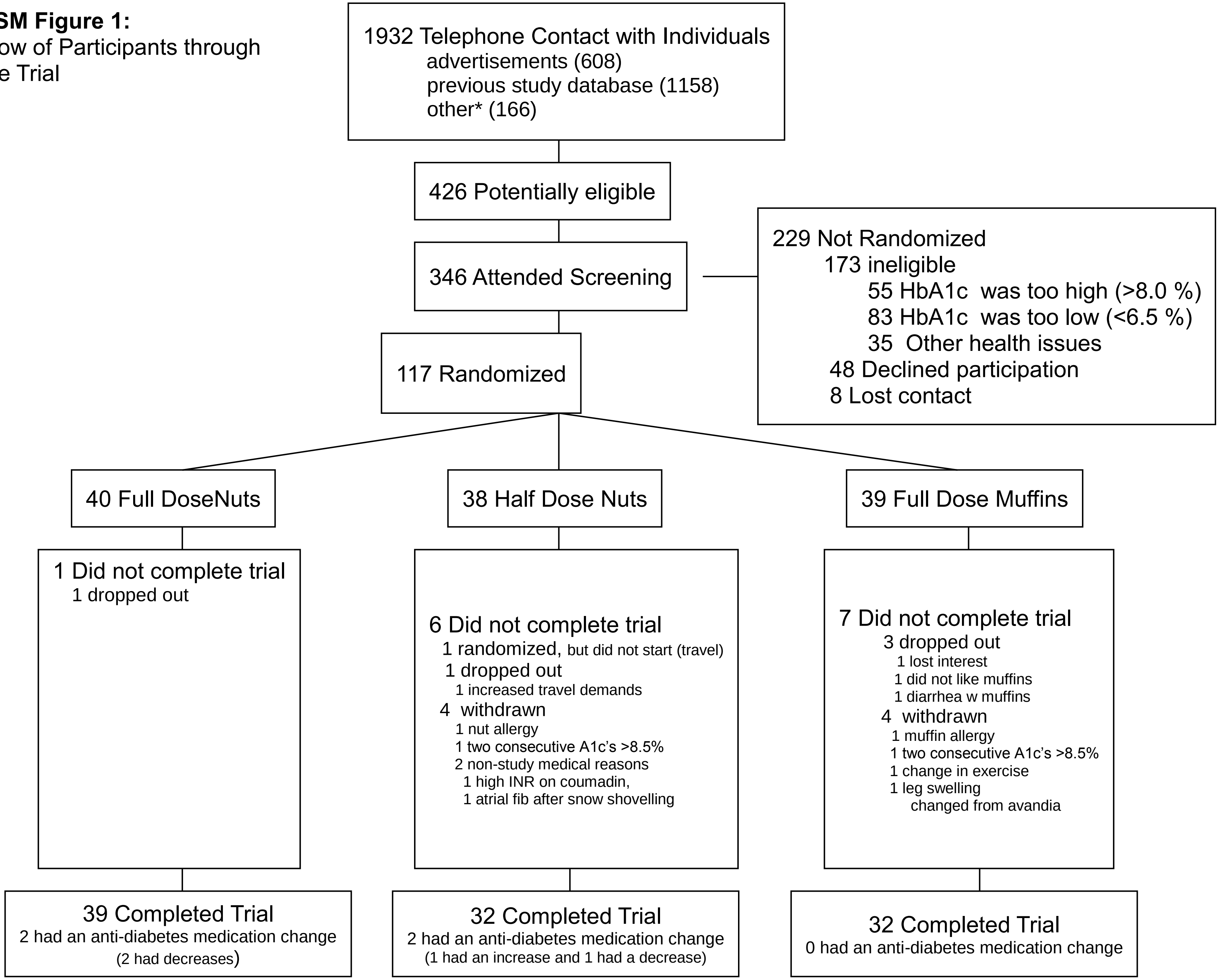

* Other includes – word of mouth, Diabetes clinic, postings in hospital, other studies, not indicated/unknown
